# History and evidence for state of the art of lymphadenectomy in esophageal cancer surgery

**DOI:** 10.1093/dote/doad065

**Published:** 2023-12-04

**Authors:** Nannet Schuring, Mark I van Berge Henegouwen, Suzanne S Gisbertz

**Affiliations:** Department of Surgery, Amsterdam UMC location University of Amsterdam, Amsterdam, The Netherlands; Cancer Treatment and Quality of Life, Cancer Center Amsterdam, Amsterdam, The Netherlands; Gastroenterology and Hepatology, Amsterdam UMC Location University of Amsterdam, Amsterdam Gastroenterology Endocrinology Metabolism, Amsterdam, The Netherlands; Department of Surgery, Amsterdam UMC location University of Amsterdam, Amsterdam, The Netherlands; Cancer Treatment and Quality of Life, Cancer Center Amsterdam, Amsterdam, The Netherlands; Department of Surgery, Amsterdam UMC location University of Amsterdam, Amsterdam, The Netherlands; Cancer Treatment and Quality of Life, Cancer Center Amsterdam, Amsterdam, The Netherlands

**Keywords:** esophageal cancer, esophagectomy, lymph node, lymphadenectomy, upper GI surgery

## Abstract

The current curative multimodal treatment of advanced esophageal cancers consists of neoadjuvant or perioperative chemo(radio)therapy followed by a radical surgical resection of the primary tumor and a 2- or 3-field lymphadenectomy. One of the most important predictors of long-term survival of esophageal cancer patients is lymph node involvement. The distribution pattern of lymph node metastases in esophageal cancer is unpredictable and depends on the primary tumor location, histology, T-stage and application of neoadjuvant or perioperative treatment. The optimal extent of the lymphadenectomy remains controversial; there is no global consensus on this topic yet. Some surgeons advocate an aggressive and extended lymph node dissection to remove occult metastatic disease, to optimize oncological outcomes. Others promote a more restricted lymphadenectomy, since the benefit of an extended lymphadenectomy, especially after neoadjuvant chemoradiotherapy, has not been clearly demonstrated, and morbidity may be reduced. In this review, we describe the development of lymphadenectomy, followed by a summary of current evidence for lymphadenectomy in esophageal cancer treatment.

## INTRODUCTION

The incidence of esophageal cancer increases, affecting 604 100 people in the year 2020 worldwide. This disease was responsible for over 544 000 deaths and accounted for over 5% of all cancer related deaths.^1^ The current curative multimodal treatment consists of neoadjuvant or perioperative chemo(radio)therapy followed by a radical surgical resection of the primary tumor and a 2- or 3-field lymphadenectomy.^2–11^ Despite improvements in treatment and perioperative care, the 5-year overall survival rarely exceeds 40%, with reported 5-year overall survival rates in landmark trials of 55.3% (cisplatin/5-fluorouracil, JCOG 9907), 45% (FLOT trial) and 47% (CROSS trial).^2^^,^^3^^,^^5–12^

Lymph node involvement is one of the most important predictors of long-term survival.^13–15^ There are various classifications for the categorization of lymph node metastases in use, differing in naming, numbering and anatomical boundaries of lymph node stations.^16–18^ The distribution pattern of lymph node metastases is unpredictable and depends on primary tumor location, histology, T-stage and application of neoadjuvant or perioperative treatment.^19–24^ A single lymph node metastasis can occur far from the primary tumor, skipping the first and even the second echelon lymph nodes, a phenomenon called skip metastasis. Identification of involved lymph nodes is necessary for effective staging and treatment. However, even with improved imaging modalities such as integrated PET-CT and EUS, clinical staging of lymph nodes is unreliable, and up to 38% of the lymph node metastases remain undetected.^25^ Current radiation fields and lymphadenectomy may not include these occult metastatic lymph nodes, and furthermore, the optimal extent of lymphadenectomy remains controversial.^18^^,^^26–28^ Some studies support an aggressive and extended lymph node dissection, as it improves accurate staging, locoregional disease control and overall survival. Other studies do not show benefit of an extended lymphadenectomy, and especially after neoadjuvant chemoradiotherapy, results are conflicting, while morbidity may be reduced following a less extended dissection.^8^^,^^29–33^

The aim of this review is to outline a brief history of lymphadenectomy and the existing heterogeneity in classification systems, to review the current evidence for the extent of lymphadenectomy and describe future developments in lymphadenectomy in esophageal cancer surgery.

## ESOPHAGEAL CANCER AND LYMPHATIC METASTASES

Esophageal cancer has two major histological subtypes: adenocarcinoma (EAC), most common in Northern and Western Europe and in North America and Oceania, and squamous cell carcinoma (ESCC), more prevalent in Asia, Africa and South America.^34–37^ Lymph node involvement of cervical, thoracic and abdominal nodes is common in both histological subtypes, even at an early disease stage and regardless of primary tumor location. This is caused by the complex organized lymphatic system surrounding the esophagus, with small branches in the lamina propria of the mucosa, and the multidirectional connections of the system.^38–40^ The lymphatic spread can follow multiple pathways: longitudinal along the submucosa to regional and non-regional lymph nodes, transversal through the muscularis propria to regional lymph nodes and perpendicular through the muscularis mucosa to the thoracic duct and the venous system.^32^ Due to this multidirectional spread, and the dense, superficial lymphatic network, metastatic lymph nodes can be found in the three compartments (cervix, thorax and abdomen) that the esophagus passes on its course toward the stomach.

## CLASSIFICATION SYSTEMS

Several classification systems exist for the categorization of lymph node metastases in esophageal cancer. The heterogeneity in classification systems makes comparison and interpretation of study results challenging, complicating exchange of current knowledge.^18^

The most used classification system for esophageal cancer in the East is the ‘Japanese Classification of Esophageal Cancer’ by the Japanese Esophageal Society (JES). The predecessor of JES, the Japanese Society of Esophageal Cancer (JSED), published the first edition in 1969. In the successive editions by the JES, modifications and changes to this classification system were made based on new evidence. For ESSC, several large cohort studies have been conducted, investigating the distribution of lymph node metastases, leading to the N-grouping in this classification system.^41^ The efficacy index, representing the prognosis of each lymph node station in relation to the primary tumor location provides recommendations which lymph node stations to resect with regard to the primary tumor location.^16^^,^^26^^,^^42^^,^^43^ The N-groups are revised in every edition, based on registry data of esophageal cancer patients in Japan.^44^

**Fig. 1:**
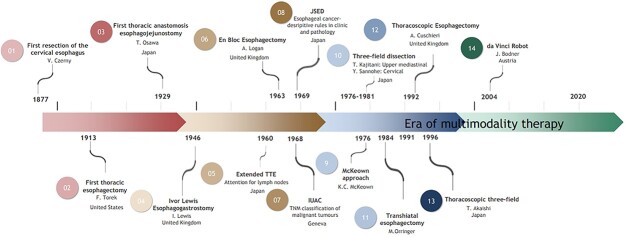
Timeline of the evolution in esophageal cancer care.

In Western countries, the most used classification system is the ‘Cancer Staging Manual’, by the American Joint Committee on Cancer (AJCC) and the Union for International Cancer Control (UICC), of which the first edition was published in 1968 (Fig. 1; 7–8).^26^^,^^45^ The most recent, 8th edition, was published in the beginning of 2017. In this manual, the N-status is based on the number rather than the location of metastatic lymph nodes, and the primary tumor location is not considered. Recommendations on which lymph node stations to resect are not provided in the ‘Cancer Staging Manual’.

The main difference between these two classification system is in the definition of the N-stage. Not only the numbers and names of these lymph node stations differ but also the anatomical boundaries of the stations. In the AJCC 8th edition, the N-category is defined by the number of metastatic regional lymph nodes, while in the JES 11th edition, regional lymph nodes are grouped into N1–N4 in five different patterns according to the primary tumor location.^18^ The most pronounced differences between the two classification systems are seen in the upper mediastinum and neck. In the AJCC classification, all cervical stations except station 1 and level VI and VII (Head and Neck AJCC cancer staging) are defined as extra-regional (M+ disease), while in the JES, cervical lymph nodes are not only classified in much more detail; they are also grouped into N1–N4 and regarded as loco-regional lymph nodes, depending on primary tumor location.

Recently, a study has been conducted to investigate which of these N-staging systems is superior in predicting esophageal cancer survival. Overall, the AJCC 8th edition N-staging classification tended to reflect the survival more precisely compared to the JES classification system. However, the difference in prediction was not significant and both staging systems are useful for predicting survival after esophagectomy. The majority of the patients (93%) in this study were diagnosed with an ESCC.^46^

In 2019, the TIGER study protocol was published, an ongoing observational cohort study investigating the distribution of lymph node metastases in esophageal cancer.^47^ This protocol contains a classification for lymph node stations in esophageal cancer in the context of the TIGER study. In this classification system, the AJCC 8th edition and the JES 11th edition are combined and simplified for research purposes. Lymph node stations 1–5 are lymph nodes in the cervical region, 6–13 are thoracic lymph node stations and 14–19 are lymph nodes located in the abdominal region. Recently, a match for the two established classification systems and the TIGER classification has been proposed, which aimed to contribute to more uniformity and to better comparison of studies about the distribution of lymph node metastasis in esophageal cancer patients (Supplementary Table 1 and Supplementary Figs 1–3).^18^^,^^47^

**Fig. 2:**
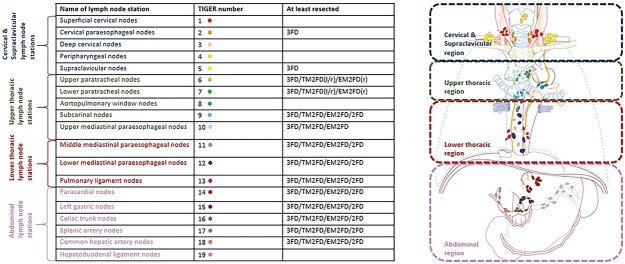
Extent of the lymphadenectomy. Overview of TIGER classification of locoregional lymph node stations of esophageal cancer. EM2F, extended mediastinal 2-field dissection; l/r, left/right; r, right; TM2FD, total mediastinal 2-field dissection; 3FD, 3-field dissection. [Partly re-used own figure from ‘Distribution of lymph node metastases in esophageal carcinoma [TIGER study]: study protocol of a multinational observational study’ by E.R.C. Hagens *et al*. published in ‘BMC Cancer’ (2019) 19:662 with permission from authors.

During the IGSC Joint Meeting in Munich in 1994, the extent of lymphadenectomy was discussed between esophageal cancers specialists and defined as ‘standard’, ‘extended’, ‘total mediastinal’ and a ‘three-field’ lymphadenectomy. The 3-field lymphadenectomy includes at least the following stations according to the JES classification system: cervical esophagus (Ce); station 101 and 104, mediastinal; station 106rec, 106pre, 106tbL and 107–112, upper abdominal; station 1–2, 3, 7, 9 and 19–20. Dissection of additional lymph node stations is performed depending on disease progression and primary tumor location. The total mediastinal 2-field includes dissection of all the lymph node stations in the 3-field dissection, except the lymph nodes in the cervical region. The extended 2-field includes all lymph nodes of the total mediastinal 2-field except the left paratracheal nodes. The standard 2-field does not include the dissection of the upper paratracheal nodes.^48^^,^^49^ A summary of which TIGER lymph node stations are resected for the different fields of lymphadenectomy extents is given in Figure 2.^18^^,^^47^

## GUIDELINES

In Japan, the most recent guideline for curative therapy of esophageal cancer recommends preoperative triplet chemotherapy with docetaxel, cisplatin plus 5-fluorouracil followed by an esophagectomy with a 3-field lymphadenectomy in case of a thoracic ESCC (stage II and III).^12^^,^^50–52^ In China, neoadjuvant chemoradiotherapy, followed by an esophagectomy with a total mediastinal 2-field dissection, is recommended for patients with mid-lower thoracic cancer and a 3-field dissection in case of suspected cervical lymph nodes metastasis or in case of upper thoracic cancer.^53^

In the West, the current ESMO (European Society of Medical Oncology) guidelines for esophageal cancer advices a transthoracic esophagectomy with an en bloc 2-field lymphadenectomy for fit patients following neoadjuvant chemoradiotherapy or perioperative chemotherapy.^54^

**Fig. 3:**
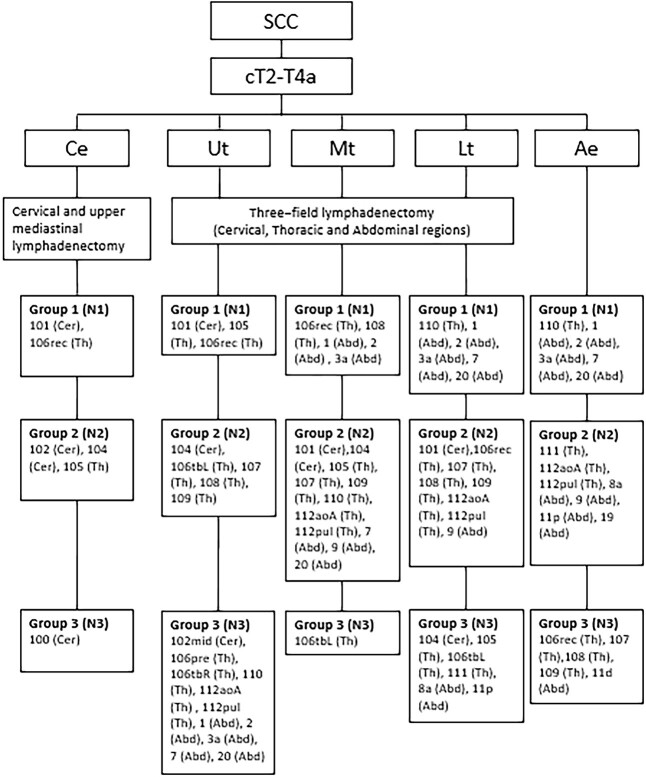
Recommended lymphadenectomy for esophageal squamous cell carcinoma. Based on the Japanese Classification of Esophageal cancer (11th edition), nodes from group 1, 2 and 3 are regarded as locoregional lymph nodes and should therefore at least be resected during lymphadenectomy.^16^ Ae, abdominal esophagus; Abd, abdominal lymph node stations; Ce, cervical esophagus; Cer, cervical lymph node stations; Lt, lower thoracic esophagus; Mt, middle thoracic esophagus; SCC, squamous cell carcinoma; Th, thoracic lymph node stations; Ut, upper thoracic esophagus;

**Fig. 4:**
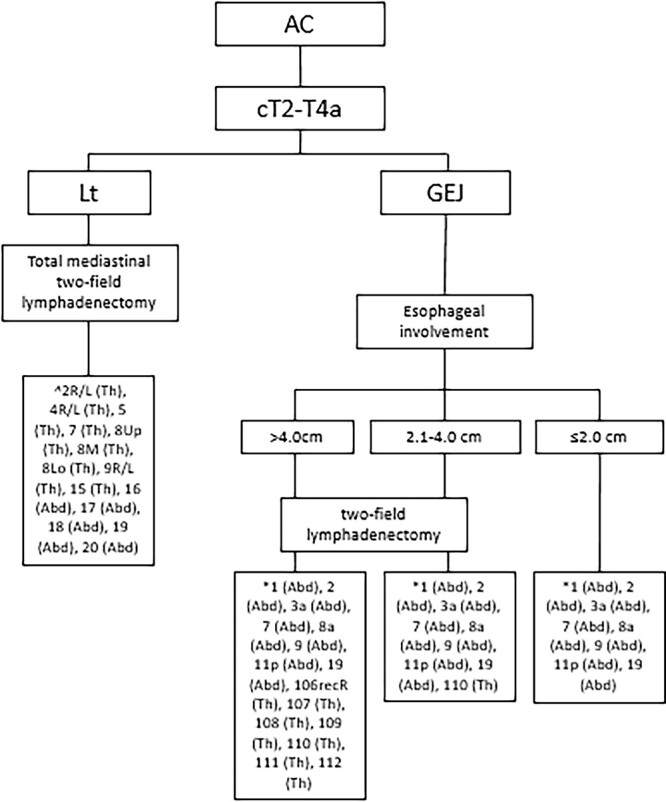
Recommended lymphadenectomy for esophageal adenocarcinoma. Based on the ESMO guidelines for esophageal cancer, 8th edition AJCC/UICC staging of cancers of the esophagus and esophagogastric junction.^27^^,^^54^^,^^67^^,^^77^ Abd, abdominal lymph node stations; AC, adenocarcinoma; GEJ, gastroesophageal junction; Lt, lower thoracic esophagus; Th, thoracic lymph node stations. *According to the JES (11th edition),^27^ ^According to the AJCC (8th edition) Amin *et al*.,^27^ Obermannová *et al*.^54^ and Hagens *et al*.^77^

For patient with an ESCC, it is generally advocated in the West to perform a mediastinal and abdominal lymphadenectomy after neoadjuvant treatment, since most of these tumors are located in the thoracic esophagus.^55^ There is, however, great variation in the level of especially the mediastinal lymphadenectomy, and dissection of cervical nodes is only performed on indication.^56^ In the Dutch guideline, neoadjuvant chemoradiotherapy followed by a transthoracic (cervical or intrathoracic anastomosis) or transhiatal esophagectomy is proposed, with the majority of the patients have a distal or esophagogastric junction adenocarcinoma. Enormous variation in extent of lymphadenectomy is observed accordingly.^57^ Also in the British guideline, the extend of the resection and lymphadenectomy is left to the discretion of the surgeon.^58^^,^^59^ Although current guidelines from the West all have multimodal treatment incorporated, the extent of lymphadenectomy is not described. A potential downfall of neoadjuvant treatment might be, besides toxicity, the radiation- or chemotherapy-induced fibrosis, which may make the dissection of lymph nodes more challenging and may increase surgical morbidity.^60^^,^^61^ Well-designed studies and trials investigating the distribution of lymph node metastases, the extent of lymphadenectomy and associated morbidity in the era of multimodal treatment are required, to make evidence-based guidelines possible (Figs 3 and 4).

## A BRIEF HISTORY OF LYMPHADENECTOMY: EAST AND WEST

### East

#### Distribution pattern of lymph node metastases

Several studies in the East have investigated the distribution of lymph node metastases in esophageal cancer (summarized in Table 1 [SCC] and Table 2 [AC]). In the systematic review by Hagens *et al*. the pooled results of 11 studies on the distribution of lymph node metastases in ESCC patients (*n* = 8543) showed that lymph node metastasis can occur in cervical, thoracic and abdominal lymph node stations, regardless of the location of the primary tumor.^41^ Seven of these studies reported data per lymph node station.^19–22^^,^^62–64^ Lymph node metastases were most frequently observed along the right recurrent nerve (60%), right cervical paraesophageal (24%) and along the left gastric artery (28%), in case of an upper, middle and lower thoracic tumor, respectively. The prevalence of lymph node metastases per region was described in six studies.^19^^,^^21^^,^^23^^,^^62^^,^^65^^,^^66^ For patients with an upper thoracic ESCC, the regions most involved were the lymph nodes in the cervical area (35%, 192/547 patients) and upper mediastinum (31%, 161/516 patients). The abdominal (20%, 787/4010 patients) and middle mediastinal (27%, 1088/4046 patients) regions were most commonly affected in case of a middle thoracic ESCC and the abdominal (29%, 466/1615patients) and lower mediastinal (25%, 434/1711 patients) regions in patients with a lower thoracic esophageal tumor.

**Table 1:** Summary of studies describing the distribution pattern of lymph node metastases for esophageal squamous cell carcinoma

Author SCC	Year	Country	Study type	Sample size	Histology	Location of primary tumor	cT (y) and pT	cN and (y)pN	Oncological treatment	Extent of lymphadenectomy	Total number of LN	Number of positive lymph nodes	Distribution of LNM/lymph node stations most frequently involved
H. Li^19^	2012	China	Retrospective	200	SCC	UTE 31 (16) MTE 137 (69) LTE 32 (16)	pT1 18 pT2 45 pT3 114 pT4 23	NR	Adjuvant chemotherapy	3FL	Mean 35.5	NR	UTE Cervical 54.8% Recurrent laryngeal nerve 41.9% Mediastinal 51.6% Abdominal 0% MTE Cervical 35% Recurrent laryngeal nerve 34.3% Mediastinal 46% Abdominal 24.8% LTE Cervical 35% Recurrent laryngeal nerve 37.5% Mediastinal 56.3% Abdominal 37.5%
S. Sharma^20^	1994	Japan	Retrospective	70	SCC	UTE 10 (14) MTE 37 (53) LTE 23 (33)	pT1 11 (16) pT2 12 (17) pT3 46 (65) pT5 1 (2)	pN0 20 (29) pN1 50 (71)	No preoperative therapy	3FL	Mean 82	208/5720 (3.6%)	Cervical 17.1% Thoracic 51.4% Abdominal 42.9%
J. Chen^20^	2009	China	Retrospective	1850	SCC	UTE 289 MTE 1381 LTE 180	pT1 109 pT2 348 pT3 1215 pT4 178	NR	No preoperative therapy	3FL	Mean 26 (range 15–71)	4350/47470 (9.2%)	UTE Cervical 49.5% Upper mediastinal 28.7% Middle mediastinal 11.4% Lower mediastinal 1.4% Abdominal 8.0% MTE Cervical 35.0% Upper mediastinal 22.4% Middle mediastinal 29.8% Lower mediastinal 6.0% Abdominal 27.2% LTE Cervical 17.2% Upper mediastinal 10.0% Middle mediastinal 25.6% Lower mediastinal 13.9% Abdominal 51.7%
S. Kosugi^22^	2013	Japan	Retrospective	86	SCC	UTE 17 (20) MTE 59 (69) LTE 10 (11)	pT1 81 (95) pT2 (5)	NR	No preoperative therapy	3FL	NR	NR	UTE Cervical 41% Upper mediastinal 24% Middle mediastinal 6% Lower Mediastinal 0% Abdominal 6%
													MTE Cervical 13% Upper mediastinal 26% Middle mediastinal 5% Lower mediastinal 6% Abdominal 27% LTE Cervical 0% Upper mediastinal 0% Middle mediastinal 20% Lower mediastinal 10% Abdominal 40%
Y. Tachimori^23^	2011	Japan	Retrospective	356	SCC	UTE 55 (15) MTE 173 (49) LTE 128 (36)	pT1 127 (35.7) pT2 40 (11.2) pT3 183 (51.4) pT4 6 (1.7)	pN0 110 (30.9) pN1 116 (32.6) pN2 81 (22.8) pN3 49 (13.8)	No preoperative therapy	3Fl	NR	NR	UTE Cervical 13.6% Upper mediastinal 54.5% Middle mediastinal 4.5% MTE Cervical 11.9% Upper mediastinal 22.4% Middle mediastinal 6.0% Lower mediastinal 9.0% Abdominal 26.9% LTE Upper mediastinal 13.2% Middle mediastinal 5.3% Lower mediastinal 5.3% Abdominal 39.5%
J. Cheng^62^	2013	China	Retrospective	1893	SCC	UTE 82 (4.3) MTE 1266 (66.9) LTE 545 (28.7)	pTis 10 (0.5) pT1 103 (5.4) pT2 345 (18.2) pT3 1173 (62) pT4 262 (13.8)	NR	No preoperative therapy	2FL or 3FL	Mean 13.4	856/1893 (45.7%)	UTE Cervical 14.6% Upper mediastinal 29.3% Middle mediastinal 8.5% Lower mediastinal 9.8% Abdominal 7.3% MTE Cervical 4.3% Upper mediastinal 5.0% Middle mediastinal 32.9% Lower mediastinal 2.5% Abdominal 14.9% LTE Cervical 2.0% Upper mediastinal 2.2% Middle mediastinal 15.4% Lower mediastinal 38.1% Abdominal 27.5%
Z. Lin^63^	2016	China	Prospective	260	SCC	CE/UTE 28 (10.8) MTE 173 (66.5) LTE 59 (22.7)	pT1 30 (11.5) pT2 44 (16.9) pT3 164 (63.1) pT4 22 (8.5)	pN0 119 (45.8) pN1 67 (25.1) pN2 54 (20.8) pN3 20 (7.7)	No preoperative therapy	2FL 107 (41.2) 3FL 153 (58.8)	Median 35 (IQR 25–46)	Median 1 (IQR 0–3)	CE/UTE (JES) Station 1 38.9% Station 2 29.3% Station 9 25.0% MTE (JES) Station 8 M 17.5% Station 1 22.9% Station 16 22.8% LTE (JES) Station 16 33.9% Station 8 L 26.7% Station 8 M 17.5%
S. Y. Park^64^	2018	Republic of Korea	Prospective	29	SCC	MTE 17 (58.6) LTE 12 (41.4)	pT1 29 (100)	cN0 25 (86.2) cN1 4 (13.8)	No preoperative therapy	Total mediastinal	Mean 54.7	NR	NR
H. Igaki^65^	2001	Japan	Retrospective	96	SCC	LTE 96 (100)	pT1 27 (28) pT2 16 (17) pT3 53 (55)	pN0 36 (38) pN1 60 (63)	No preoperative therapy	3FL	NR	NR	Cervical 8.3% Upper mediastinal 17.7% Middle mediastinal 18.8% Lower mediastinal 49.0% Abdominal 51.0%
Y. Dong^66^	2015	China	Retrospective	3587	SCC	UTE 189 (5.3) MTE 1837 (51.2) LTE (43.5)	pT1 435 (14) pT2 935 (25) pT3 1993 (55) pT4 225 (6)	pN0 2223 (62) pN1 1233 (34) pN2 98 (3) pN3 33 (1)	No preoperative therapy	2FL 3115 (86.8) 3FL 472 (13.2)	Mean 20 (range 16–50)	2870/71 740 (4%)	UTE Cervical 11.1% Upper mediastinal 15.6% Middle mediastinal 2.2% Lower mediastinal 2.2% Abdominal 1.1% MTE Cervical 5.9% Upper mediastinal 5.2% Middle mediastinal 16.6% Lower mediastinal 4.1% Abdominal 15.3% LTE Cervical 1.8% Upper mediastinal 1.4% Middle mediastinal 14.4% Lower mediastinal 20.3% Abdominal 31.4%
H. Miyata^69^	2019	Japan	Prospective	561	SCC	MTE 294 (52.4) LTE 267 (47.6)	pT0 37 (6.6) pT1 112 (20) pT2 87 (15.5) pT3 306 (54.5) pT4 19 (3.4)	pN0 170 (30.3) pN1 181 (32.4) pN2 106 (18.9) pN3 104 (18.5)	NCRT (2 courses of ACF or DCF)	2FL 242 (43.1) 3FL 319 (56.8)	Mean 70.4	Number of positive nodes 4.4 (± 10)	NR
B. Li^72^	2020	China	RCT	400	SCC	MTE 281 (70.3) LTE 119 (29.8)	pT1 102 (25.5) pT2 96 (24) pT3 178 (44.5) pT4 24 (6)	pN0 205 (51.3) pN1 104 (26) pN2 60 (15) pN3 31 (7.8)	No preoperative therapy	3FL 2FL	3FL: median 37 (IQR 30–49) 2FL: median 24 (18–30)	NR	3FL Cervical 21.5% Thoracic 34.5% Abdominal 31.0% 2FL Thoracic 36.5% Abdominal 28.5%

**Table 2:** Summary of studies describing the distribution pattern of lymph node metastases for esophageal adenocarcinoma

Author AC	Year	Country	Study type	Sample size	Histology	Location of primary tumor	cT (y) and pT (%)	cN and (y)pN (%)	Oncological treatment (%)	Extent of lymphadenectomy	Total number of LNs	Number of positive lymph nodes	Distribution of LNM/lymph node stations most frequently involved
Siewert^76^	2006	Germany	Prospective	1602	AC	AEG type 1621 AEG type 2485 AEG type 3496	AEG type 1 pT1 213 (34.3) pT2 155 (24.9) pT3 223 (35.9) pT4 30 (4.9) AEG type 2 pT1 79 (16.2) pT2 270 (55.7) pT3 108 (21.7) pT4 31 (6.4) AEG type 3 pT1 41 (8.3) pT2 173 (35.1) pT3 204 (41.1) pT4 77 (15.5)	AEG type 1 pN0 299 (48.1) pN1 (322 (51.9) AEG type 3 pN0 169 (34.8) pN1 166 (34.2) pN2 120 (24.7) pN3 30 (6.3) AEG type 3 pN0 110 (22.2) pN1 132 (26.6)) pN2 200 (40.3) pN3 54 (10.9)	Primary resection 402 (64.7) Neoadjuvant chemotherapy 219 (35.3)	NR	NR	NR	AEG type 1 left paracardial region (50%) right paracardial region (53%) lower posterior mediastinum (50%) AEG type 2 left paracardial region (67%) right paracardial region (63%) lesser curvature (66%) AEG type 3 left paracardial region (49%) right paracardial region (52%) lesser curvature (85%)
Hagens^77^	2020	Netherlands	Prospective	50	AC	MTE 3 (6) LTE 47 (95)	cT1 1 (2) cT2 11 (22) cT3 38 (76) ypT0 6 (12) ypT1 11 (22) ypT2 6 (12) ypT3 27 (54)	cN0 12 (24) cN1 23 (46) cN2 12 (26) cN3 2 (4) ypN0 20 (40) ypN1 11 (22) ypN2 11 (22) ypN3 8 (16)	Neoadjuvant chemoradiotherapy CROSS 46 (92) TRAP 4 (8)	2FL	Median 37 (IQR 26–43)	Median 3 (IQR 1–8)	Paraesophageal lymph nodes (28%) Left gastric artery nodes (24%) Celiac trunk lymph nodes (18%) Left paracardial nodes (16%)

AE, abdominal esophagus; AC, adenocarcinoma; LTE, lower thoracic esophagus; MTE, middle thoracic esophagus; NR, not reported; SCC, squamous cell carcinoma; UTE, upper thoracic esophagus; 2FL, 2-field lymphadenectomy; 3FL, 3-field lymphadenectomy.

Also, the dissemination pattern in gastro-esophageal junction cancers, mainly adenocarcinoma, was studied in a recent prospective Japanese nationwide study, including 363 patients (AC 91.5%, SCC 8.5%). The minority of the included patients received neoadjuvant treatment (33.3%). The observed involvement of the right recurrent laryngeal nerve station was 10.7% (3/28) when esophageal involvement was >4.0 cm.^67^ These results led to the strong recommendation for dissection of this upper mediastinal station in patients with esophageal involvement of more than 4.0 cm.

In two recently published studies, the distribution of lymph node metastases after multimodal therapy was investigated.^68^^,^^69^ In the study of Hamai *et al*., published in 2020, lymph node metastases were present in 86 of the 184 (46.7%) ESCC patients treated with neoadjuvant chemoradiation and surgery and positive lymph nodes were found in all three compartments with the majority within the radiation field.^68^ Residual metastases were mainly located in the upper mediastinal and upper abdominal lymph node stations, in case of tumors located in the upper and lower thoracic esophagus, respectively. In the prospective study by Miyata *et al*., including 561 patients with middle or lower thoracic esophageal cancer (95.2% ESCC) treated with neoadjuvant chemotherapy followed by an esophagectomy, the efficacy index was studied.^69^ Irrespective of primary tumor location, the efficacy index was high for cardiac and recurrent laryngeal nerve lymph nodes, and the efficacy index did not vary according to primary tumor response to neoadjuvant therapy.

#### Development of 3-field lymphadenectomy

Until the 1970s, lymphadenectomy was limited to stations in the lower mediastinum and the abdomen, since an esophagectomy was a procedure with a high complication and mortality rate.^49^ Before 1924, a mortality rate >95% has been described. In that period, modern surgical techniques, instruments and anesthetic techniques were not yet available, let alone lymphadenectomy. The establishment of 3-field lymphadenectomy in the East is based on some of the following studies. T. Kajitani and I. Kinoshita (1976, Fig. 1, 10) were the first to resect upper mediastinal nodes and reported an incidence of involvement of lymph nodes along the recurrent laryngeal nerves in almost one-third of the esophageal cancer patients (30/95).^70^ After publication of other small cohort studies, in 1994, Akiyama *et al*. published their cohort study on systematic lymph node dissection in 717 patients with a thoracic ESCC and found a significant 5-year overall survival benefit for patients in which a 3-field dissection (55.0%) compared to a 2-field (38.3%) lymphadenectomy was performed (*P* = 0.001).^71^ Since then, consensus in the East exists for a 3-field lymphadenectomy. This is strengthened by the outcomes of a recent randomized controlled trial comparing 2-field versus 3-field lymphadenectomy in patients with a squamous cell carcinoma of the middle or distal esophagus, who did not receive neoadjuvant chemo(radio)therapy.^72^ Postoperative morbidity and mortality were comparable between both groups, with a higher lymph node yield that led to stage migration in the 3-field group. Long-term survival data of this study, however, have to be awaited. Furthermore, the results of a recently published meta-analysis on the role of prophylactic dissection of the supraclavicular nodes in patients with a thoracic ESCC showed that, although a trend toward improved overall survival in favor of prophylactic dissection was observed, no significant difference in long- or short-term outcomes were found.^73^

In a meta-analyses by Ma *et al*. (20 included studies, >7000 patients, no information on histological subtype) a survival benefit for patients with a 3-field lymphadenectomy, but more complications, were found compared to patients with a 2-field lymphadenectomy.^74^ A comparable survival benefit and no significant differences in postoperative morbidity or mortality was observed in a meta-analysis published in 2022 by Bona *et al*. (14 included studies, 3431 ESCC patients, conducted in the East [Japan, China and South Korea]).^75^

In most described studies, (neo)adjuvant chemo (radio)therapy was either not applied or inconsistently and heterogeneously reported and in neither meta-analysis, subgroup analyses were performed for (neo)adjuvant therapy. Since neoadjuvant therapy is currently the standard of care, these studies may have limited value in the present era.

### West

#### Distribution pattern of lymph node metastases

R.J. Siewert and J.H. Stein were the first to report on the pattern of lymphatic spread according to the Siewert classification in large series of patients (*n* = 1602) with an adenocarcinoma of the esophagogastric junction.^76^ They published a map of lymph node metastases by Siewert class of esophagogastric junction cancer. The incidence of involved stations differed markedly by Siewert class, although in all three types, lymph node metastases were observed in the mediastinum and abdomen. In a study by Hagens *et al*., an incidence of 60% lymph node metastases was observed in patients (*n* = 50) with a mid- or distal esophageal adenocarcinoma all after neoadjuvant chemoradiotherapy.^77^ High-risk zones were lymph nodes in the paraesophageal (28%), left gastric artery (24%), celiac trunk (18%) and left paracardial (16%) stations. The majority of the metastatic lymph nodes was located inside the radiation field (52%), thereby suggesting that radical surgery remains essential in the curative treatment of esophageal cancer after neoadjuvant chemoradiotherapy. Findings of these studies are summarized in Table 1 [SCC] and Table 2 [AC].

#### Development of lymphadenectomy

In the West, Orringer, McKeown and Ivor Lewis can be seen as the founding fathers of the current three major operative approaches of the esophagus: the 2-stage transthoracic esophagectomy (Ivor Lewis), the 3-stage transthoracic esophagectomy (McKeown) and the transhiatal esophagectomy (Orringer). Ivor Lewis was the first to perform a two-stage transthoracic esophagectomy with esophagogastrostomy in the thorax in 1946 (Fig. 1; 4).^78^ K. C. McKeown described a three-stage esophagectomy in 1976 (Fig. 1; 6). He also described that the presence of lymph node metastases depended on the site of the primary tumor: when located distally, the celiac trunk, left gastric artery, splenic artery and pancreatic lymph nodes should be resected.^79^ M. Orringer was the first to report on a series of transhiatal esophagectomies in 1984 (Fig. 1; 11).^80^

Contradictory to the East, no standardized procedure for the extent of lymph node dissection per primary tumor location or other tumor characteristics exists. Lacking knowledge on the distribution of lymph nodes metastases and on the prognostic value of resecting different lymph node stations in patients with an EAC makes an evidence-based guideline not yet possible. Currently, for distal esophageal or gastroesophageal junction AC, the extent of the dissection differs considerably, especially with regard to the upper mediastinum and neck.

In 2008, Peyre *et al*. investigated the relationship between the extent of the lymphadenectomy and survival in 2303 patients from nine specialty centers (five from Europe, three from North America and one from Asia), who had undergone an R0 esophagectomy for an EAC or ESCC of the esophagus without preoperative chemo(radio)therapy. Their results showed that the number of lymph nodes removed is an independent predictor of survival and that 23 regional nodes had to be removed to maximize the survival benefit.^81^

Lagergren *et al*. published a retrospective study in 2015, where they investigated survival with regard to lymph node yield in 606 patients (83.5% EAC, majority of the patients received preoperative chemotherapy [391/606, 64.5%]).^82^ No survival benefit for an increased lymph node yield was found, when grouped as 0–10, 11–14, 15–20 or 21–52 nodes. However, there were more well-differentiated tumors in the lower quartile groups and more poorly differentiated tumors in the higher quartile groups. Furthermore, a higher rate of pT3–T4 tumors were included in the higher quartile groups, and a complete pathologic response was overrepresented in the lower two quartiles of lymphadenectomy. Additionally, in this study details on the clinical and pathological N-stage were not provided, the surgical procedures were not described nor standardized and median lymph node count was only 14, with in over 50% of patients less than 15 resected lymph nodes. Also, a high rate of R+ resections was performed (44%), which may have contributed to the similar overall survival rates in patient groups.

Talsma *et al*. found comparable results.^31^ The number of resected lymph nodes was not associated with survival in patients treated with neoadjuvant chemoradiotherapy in the CROSS-trial cohort. Also in this study, the median number of resected lymph nodes was low with a median of only 14 (IQR 9–21) resected nodes in the nCRT plus surgery group, with a median of 0 (0–1) positive nodes, and a median of 18 (IQR 12–27) resected nodes in the surgery alone group, with a median of 2 (IQR 1–6) positive nodes. The R0 resection rate in this study was 92% in the nCRT plus surgery group and only 69% in the surgery alone group, while only patients with a cT1-N1M0 or T2-3N0-1M0 (maximum tumor length 8 cm) carcinoma of the esophagus or esophagogastric junction were included. In this study, lymphadenectomy and surgical procedure (transhiatal or transthoracic procedures) were not standardized and there was no surgical quality assurance protocol.^25^

On the contrary, it has been shown that a high incidence of lymph node metastases is still observed after neoadjuvant chemoradiotherapy,^77^ and several recently published studies support a radical lymphadenectomy even after neoadjuvant therapy.^83–85^

In the study of Henckens *et al*., 519 of 585 EAC patients (88.7%) received neoadjuvant chemoradiation, and a lymph node yield of >30 was associated with more resected positive lymph nodes, upstaging and a superior disease-free survival, without increasing morbidity.^83^ Sihag *et al*. found a survival benefit for a more extensive lymph node yield in 778 patients with EAC treated with neoadjuvant chemoradiotherapy followed by an esophagectomy, regardless of treatment response.^84^In a meta-analysis by Visser *et al*., the association between extent of the lymphadenectomy and overall survival in patients treated with and without neoadjuvant therapy was investigated. The results of the pooled analysis of seven studies of patients receiving neoadjuvant therapy followed by esophagectomy showed that a high lymph node yield was associated with superior overall survival (HR = 0.82; 95% CI = 0.73–0.92; *P*-value < 0.01)^85^ (Figs 3 and 4).

## FUTURE PERSPECTIVES

### Distribution of lymph node metastases

An ongoing study investigating the distribution of lymph node metastases is the TIGER study; this is a worldwide multicenter prospective study (NCT03222895), with the aim to determine the distribution pattern of lymph nodes metastases in esophageal cancer in relation to tumor histology, tumor location, tumor invasion depth and neoadjuvant therapy.^47^ With the results from the TIGER study, it will be possible to create a map with an efficacy index of each lymph nodes station with regard to characteristics of the primary tumor. The study results may contribute to global consensus on the optimal extent of lymphadenectomy in esophageal cancer patients. Because of the observational nature of the study, the extent of lymphadenectomy is not prescribed in the TIGER study. To ensure reliable interpretation of the TIGER results according to the actually performed lymphadenectomy, a Surgical Quality Assessment (SQA) tool will be developed, utilized and validated to examine the quality of surgical performance within the TIGER study (KWF_2021-2_14207). Additionally, a surgical artificial intelligence (AI)–based automatic video assessment concept will be developed (TIGER-SQA-AI).

### Staging with MRI with USPIOS

Targeting all metastatic lymph nodes to be included in the radiation field of neoadjuvant treatment and in the lymphadenectomy will improve locoregional tumor control and possibly survival. Current diagnostic modalities are not accurate to visualize all involved lymph nodes. A potentially promising technique to be able to more accurately detect lymph node metastasis could be MRI with a higher signal-to-noise ratio of a 3T system. Until now, MRI for esophageal cancer has been limited to assessment with a low magnetic field strength (1.5T), where the small size of the lymph nodes and the presence of physiological motion, pose major challenges. 3T MRI with the use of USPIOs (ultra-small superparamagnetic iron oxides) bears great promise for the detection of lymph node metastases, as has been shown for prostate cancer.^86^^,^^87^ On iron-sensitive magnetic resonance imaging, normal nodes lose the MR signal and are black, whereas metastatic lymph nodes remain white. This technique may also be applicable in esophageal cancer and substantially improve the individualization of esophageal cancer treatment.^88^

### Endoscopic resection with sentinel node navigation surgery in T1 esophageal cancer

Patients with a high-risk T1b esophageal carcinoma (i.e. deep submucosal invasion >500 μm, lymphovascular invasion and/or poor tumor differentiation), in whom the primary tumor is radically resected, still have a risk up to 45% of lymph node metastases.^89–95^ In these patients, a sentinel node procedure could be performed, using a dual tracer of technetium-99m nanocolloid and indocyanine green (ICG). The SNAP study group investigated this in patients with a high-risk T1 EAC and found that SNs were identified in all patients and there was a high concordance between the preoperative detection with lymphoscintigraphy/SPECT-CT and the intraoperative SN detection.^39^ In the first 10 patients (5 with technetium-99m nanocolloid only and 5 with a dual tracer), esophagectomy was still performed and revealed no retained lymph node metastases.^95^^,^^96^In the next 10 patients, esophagectomy was not performed. In two patients, a tumor-positive sentinel lymph node was resected, both patients did not undergo esophagectomy and are currently followed up^95^^,^^96^ (NTR: Trial NL8100, NL 5113, NL8558, NL7167). The use of SNNS in SCC is described by Kitagawa *et al*. and shows feasibility of this technique for patients with T1 SCC.^90^

### ICG-guided lymphadenectomy

Several studies have evaluated the feasibility and usefulness of ICG-guided lymphadenectomy using near-infrared imaging in gastric cancer, esophageal and gastro-esophageal cancer, with promising results.^97–101^ A randomized controlled trial (*n* = 266) by Chen *et al*. investigated the safety and efficacy of ICG in laparoscopic radical gastrectomy with D2 resection for patients with gastric adenocarcinoma and found that this technique can improve the number of lymph nodes harvested and reduce lymph node noncompliance compared to the conventional dissection.^98^ This technique may also apply to lymphadenectomy in esophagectomy and is currently under investigation.

## MINIMALLY INVASIVE ESOPHAGECTOMY

A. Cuschieri was the first to describe their experiences of performing an esophagectomy through a right thoracoscopic approach Cuschieri (1992, Fig. 1, 12).^102^ This procedure was followed by the introduction of a minimally invasive laparoscopic transhiatal procedure by Sadanaga *et al*.^103^ Over the past decades, minimally invasive esophagectomy has become increasingly utilized, as lower complication rates and shorter hospital stay have been described following MIE.^5^^,^^104–107^ In the TIME trial, 115 patients with resectable intrathoracic esophageal carcinoma were randomized between open and minimally invasive esophagectomy. Short-term benefits for minimally invasive esophagectomy (MIE, less pulmonary complications, shorter hospital stay and better QoL), with similar rates of overall and disease-free survival, were found.^5^^,^^104^ Similar results, with lower morbidity and comparable survival in favor of the less invasive approach, were found in the MIRO trial (*n* = 207), comparing a hybrid with an open esophagectomy.^105–107^ Currently, an ongoing randomized phase III trial (JCOG1409) investigates whether the thoracolaparoscopic approach is non-inferior to open esophagectomy in terms of overall survival. This trial aims to accrue 300 patients from Japanese institutions. The last follow-up is anticipated in 2027, with subsequent publication of the results thereafter.^108^ Especially for total MIE, a long proficiency gain curve has been described, leading to learning associated morbidity in patients.^109–113^ This learning curve is shorter when learning in a high-volume center.^109–113^ Robotic-assisted minimally invasive esophagectomy (RAMIE) may overcome some of the challenges, by offering improved visualization by 3D images and increased augmentation, instrument articulation, superior ergonomics and tremor filtration. The ongoing ROBOT-2 trial, comparing RAMIE with conventional MIE, will investigate whether RAMIE will be superior with regard to lymphadenectomy to conventional MIE in esophagectomy for cancer.^114^ Other developments in robotics, such as integrated fluorescence cameras and CT or MR images creating an augmented reality, may lead to more accurate lymph node dissection.^115^ Furthermore, single-port transcervical esophagectomy is increasingly being studied, both with conventional MIE as with RAMIE. Preclinical cadaver studies are now followed by the first studies in patients and confirming safety and feasibility, although large studies are still lacking.^116^^,^^117^ Besides the da Vinci, new robotic systems such as the Hinotori, the Hugo and the Versius® are becoming available for clinical practice, which will make robotic surgery more widely available.

## CONCLUSION

*In conclusion,* worldwide, several classification systems for lymph node staging are in use, and the extent of lymphadenectomy is not standardized. More evidence with regard to the distribution pattern of lymph node metastases and the effect of the extent of lymphadenectomy on survival in the era of neoadjuvant therapy is needed, which will result in improved staging and individualized treatment of esophageal cancer patients.

## Supplementary Material

supp_doad065
