# Supplementary material for: History and evidence for state of the art of lymphadenectomy in esophageal cancer surgery
Source: Dis Esophagus. 2023 Dec 4;37(4):doad065. doi: 10.1093/dote/doad065 (PMC10987971; doi:10.1093/dote/doad065)
Supplement: supp_doad065 [file supp_doad065.docx]

**Supplementary material**


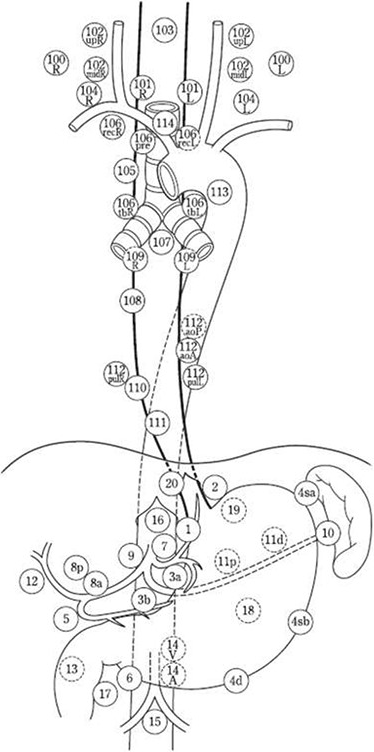


**Supplementary figure 1. JES classification (11th edition) station numbers of regional lymph** nodes**.** *[This figure is re-used. The original source: Japanese Classification of Esophageal Cancer 11^th^ Edition by the Japan Esophageal Society. (CC by 4.0* [*https://creativecommons.org/licenses/by/4.0/legalcode*](https://creativecommons.org/licenses/by/4.0/legalcode)*)]*

***
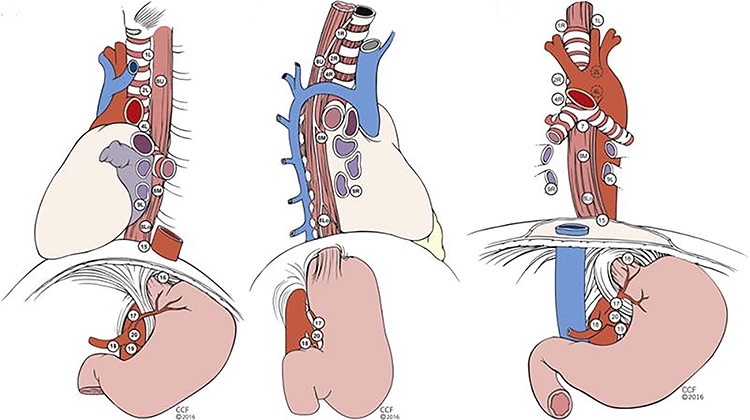
***

**Supplementary figure 2. AJCC classification (8^th^ edition) Regional lymph node maps for** esophageal cancer [*This figure is re-used. The original source: Cancer of the Esophagus and Esophagogastric Junction: An eight edition staging primer by Thomas W. Rice et al. published in Journal of thoracic Oncology 2016 with permission from Elsevier. License number: 5651300328439]*

*
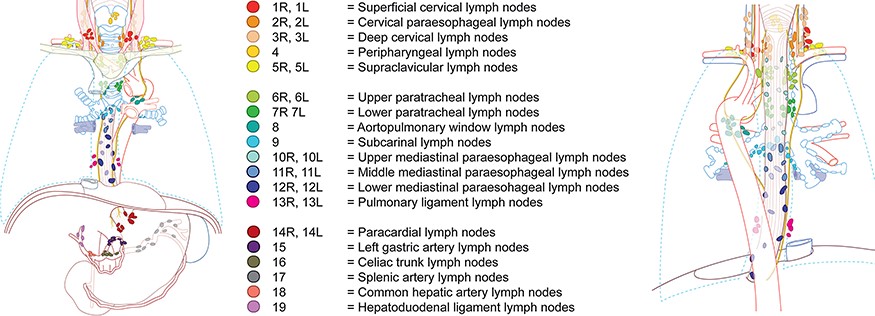
*

**Supplementary figure 3. TIGER classification (1^st^ edition) station numbers and naming of regional lymph nodes.** *[This figure is re-used. The original source:  Distribution of lymph node metastases in esophageal carcinoma [TIGER study]: study protocol of a multinational observational study by Hagens et al. Published in BMC Cancer 2019 . (CC0 1.0)]*

***Supplementary table 1.* *Proposed match of the AJCC, JES and TIGER classification system for lymph node stations in esophageal cancer***

| **JES (11^th^ edition)** |  | | | **AJCC (8^th^ edition)** |  | **TIGER (1^st^ edition)** |  |
| --- | --- | --- | --- | --- | --- | --- | --- |
| **Number** | **Name** | | | **Number** | **Name** | **Number** | **Name** |
| ***Cervical Lymph node stations*** | |  | |  |  |  |  |
|  |  | | | IA* | Submental lymph nodes |  |  |
|  |  | | | VII* | Superior mediastinal lymph nodes |  |  |
| 100 spf | Superficial cervical lymph nodes | | |  |  | 1 | Superficial cervical lymph nodes |
| 100 sm | Submandibular lymph nodes | | | IB* | Submandibular lymph nodes | 1 | Superficial cervical lymph nodes |
| 100 tr | Cervical pretracheal lymph nodes | | | VI* | Anterior compartment lymph nodes | 1 | Superficial cervical lymph nodes |
| 100 ac | Accessory nerve lymph nodes | | | VA & VB* | Posterior triangle lymph nodes | 1 | Superficial cervical lymph nodes |
| 101 | Cervical paraesophageal lymph nodes | | | 1 R/L (IV*) | Lower cervical paratracheal lymph nodes | 2 | Cervical paraesophageal lymph nodes |
| 102 up | Upper deep cervical lymph nodes | | | IIB* | Upper jugular lymph nodes | 3 | Deep cervical lymph nodes |
| 102 mid | Middle deep cervical lymph nodes | | | III* | Middle jugular lymph nodes | 3 | Deep cervical lymph nodes |
| 103 | Peripharyngeal lymph nodes | | | IIA & III* | Upper jugular & Middle jugular lymph nodes | 4 | Peripharyngeal lymph nodes |
| 104 | Supraclavicular lymph nodes | | | IV & VB* | Lower jugular & posterior triangle lymph nodes | 5 | Supraclavicular lymph nodes |
| ***Thoracic Lymph Node stations*** | | |  |  |  | |  |
| 105 | Upper thoracic paraesophageal lymph nodes | | | 8up | Upper thoracic paraesophageal lymph nodes | 10 | Upper mediastinal paraesophageal lymph nodes |
| 106 recL | Left recurrent nerve lymph nodes | | | 2 L | Left upper paratracheal lymph nodes | 6 L | Left upper paratracheal lymph nodes |
| 106 recR | Right recurrent nerve lymph nodes | | | 2 R | Right upper paratracheal lymph nodes | 6 R | Right upper paratracheal lymph nodes |
| 106 pre | Pretracheal lymph nodes | | | 4 R | Right lower paratracheal lymph nodes | 7 R | Right lower paratracheal lymph nodes |
| 106 tbL | Tracheobronchial lymph nodes | | | 4 L | Left lower paratracheal lymph nodes | 7 L | Left lower paratracheal lymph nodes |
| 106 tbR | Right tracheobronchial lymph nodes | | | 4 R | Right lower paratracheal lymph nodes | 7 R | Right lower paratracheal lymph nodes |
| 107 | Subcarinal lymph nodes | | | 7 | Subcarinal lymph nodes | 9 | Subcarinal lymph nodes |
| 108 | Middle thoracic paraesophageal lymph nodes | | | 8m | Middle thoracic paraesophageal lymph nodes | 11 | Middle mediastinal paraesophageal lymph nodes |
| 109 | Main bronchus lymph nodes | | | 10*®* | Tracheobronchial lymph nodes | 9 | Subcarinal lymph nodes |
| 110 | Lower thoracic paraesophageal lymph nodes | | | 8lo | Lower thoracic paraesophageal lymph nodes | 12 | Lower mediastinal paraesophageal lymph nodes |
| 111 | Supradiaphragmatic lymph nodes | | | 15 | Diaphragmatic lymph nodes | 12 | Lower mediastinal paraesophageal lymph nodes |
| 112 ao(A/P) | thoracic paraaortic lymph nodes (Anterior/Posterior) | | | 8m & 8lo | Middle thoracic paraesophageal lymph nodes & Lower thoracic paraesophageal lymph nodes | 11 & 12 | Middle mediastinal paraesophageal lymph nodes /Lower mediastinal paraesophageal lymph nodes |
| 112 pul | Pulmonary ligament lymph nodes | | | 9 R/L | Pulmonary ligament lymph nodes | 13 R/L | Pulmonary ligament lymph nodes (L/R) |
| 113 | Ligamentum arteriosum lymph nodes (Botallo lymph nodes) | | | 5^#^ | Aortopulmonary lymph nodes | 8 | Aortopulmonary window lymph nodes |
| 114 | Anterior mediastinal lymph nodes | | |  |  |  |  |

| **JES (11^th^ edition)** |  | | **AJCC (8^th^ edition)** |  | **TIGER (^1st^ edition)** |  |
| --- | --- | --- | --- | --- | --- | --- |
| **Number** | **Name** | | **Number** | **Name** | **Number** | **Name** |
| ***Abdominal Lymph node stations*** | |  |  | |  |  |
| 1 | Right paracardial lymph nodes | | 16 | Paracardial lymph nodes | 14 R | Right paracardia lymph nodes |
| 2 | Left paracardial lymph nodes | | 16 | Paracardial lymph nodes | 14 L | Left paracardia lymph nodes |
| 3 a | Lesser curvature lymph nodes | | 17 | Left gastric lymph nodes | 15 | Left gastric lymph nodes |
| 3 b | Lesser curvature lymph nodes | |  |  |  |  |
| 4 sa | Lymph nodes along the short gastric vessels | |  |  |  |  |
| 4 sb | Lymph nodes along the left gastroepiploic artery | |  |  |  |  |
| 4 d | Lymph nodes along the right gastroepiploic artery | |  |  |  |  |
| 5 | Suprapyloric lymph nodes | |  |  |  |  |
| 6 | Infrapyloric lymph nodes | |  |  |  |  |
| 7 | Lymph nodes along the left gastric artery | | 17 | Left gastric lymph nodes | 15 | Left gastric lymph nodes |
| 8 a | Lymph nodes along the common hepatic artery (anterosuperior group) | | 18 | Common hepatic lymph nodes | 18 | Common hepatic artery lymph nodes |
| 8 p | Lymph nodes along the common hepatic artery (posterior group) | | 18 | Common hepatic lymph nodes | 18 | Common hepatic artery lymph nodes |
| 9 | Lymph nodes along the celiac artery | | 20 | Celiac lymph nodes | 16 | Celiac trunk lymph nodes |
| 10 | Lymph nodes at the splenic hilum | |  |  | 17 | Splenic artery lymph nodes |
| 11 p | Lymph nodes along the proximal splenic artery | | 19 | Splenic lymph nodes | 17 | Splenic artery lymph nodes |
| 11 d | Lymph nodes along the distal splenic artery | | 19 | Splenic lymph nodes | 17 | Splenic artery lymph nodes |
| 12 | Lymph nodes in the hepatoduodenal ligament | |  |  | 19 | Hepatoduodenal ligament lymph nodes |
| 13 | Lymph nodes on the posterior surface of the pancreatic head | |  |  |  |  |
| 14 A | Lymph nodes along the superior mesenteric artery | |  |  |  |  |
| 14 V | Lymph nodes along the superior mesenteric vein | |  |  |  |  |
| 15 | Lymph nodes along the middle colic artery | |  |  |  |  |
| 16 a1 | Lymph nodes in the aortic hiatus | |  |  |  |  |
| 16 a2 | Lymph nodes around the abdominal aorta | |  |  |  |  |
| 16 b1 | Lymph nodes around the abdominal aorta | |  |  |  |  |
| 16 b2 | Lymph nodes around the abdominal aorta | |  |  |  |  |
| 17 | Lymph nodes on the anterior surface of the pancreatic head | |  |  |  |  |
| 18 | Lymph nodes along the inferior margin of the pancreas | |  |  |  |  |
| 19 | Infradiaphragmatic lymph nodes | | 16 | Paracardial lymph nodes | 14 R/L | Paracardia lymph nodes |
| 20 | Lymph nodes in the esophageal hiatus of the diaphragm | | 16 | Paracardial lymph nodes | 14 R/L | Paracardia lymph nodes |

*® AJCC Lung cancer staging (8^th^ edition)*

** AJCC Head and neck cancer staging (8^th^ edition)*

*^#^ AJCC Cancer staging manual: Esophagus and esophagogastric junction (7^th^ edition)*

***Supplementary table 1.*** *Proposed match of the AJCC, JES and TIGER classification system for lymph node stations in esophageal cancer*

*This table is re-used. The original source: A proposal for uniformity in classification of lymph node stations in esophageal cancer. Schuring et al. Published in Diseases of the Esophagus 2021. (CC0 1*
